# PTP1B mediates the inhibitory effect of MFGE8 on insulin signaling through the β5 integrin

**DOI:** 10.1016/j.jbc.2024.105631

**Published:** 2024-01-08

**Authors:** Ritwik Datta, Dibyanti Mukherjee, Michael J. Podolsky, Christopher D. Yang, Diana L. Alba, Sukhmani Singh, Thomas D. Arnold, Suneil Koliwad, Carlos O. Lizama, Kamran Atabai

**Affiliations:** 1 Cardiovascular Research Institute, University of California, San Francisco, California, USA; 2 Department of Pediatrics, University of California, San Francisco, California, USA; 3 Diabetes Center, University of California, San Francisco, California, USA; 4 Division of Endocrinology, Department of Medicine, University of California, San Francisco, California, USA; 5 Lung Biology Center, University of California, San Francisco, California, USA

**Keywords:** insulin recpetor, integrins, MFGE8, insulin signaling, insulin resistance

## Abstract

Integrins are cell adhesion receptors that dimerize to mediate cell–cell interactions and regulate processes, including proliferation, inflammation, and tissue repair. The role of integrins in regulating insulin signaling is incompletely understood. We have previously shown that binding of the integrin ligand milk fat globule epidermal growth factor like 8 (MFGE8) to the αvβ5 integrin promotes termination of insulin receptor signaling in mice. Upon ligation of MFGE8, integrin β5 complexes with the insulin receptor beta (IRβ) in skeletal muscle, resulting in dephosphorylation of IRβ and reduction of insulin-stimulated glucose uptake. Here, we investigate the mechanism by which the interaction between β5 and IRβ impacts IRβ phosphorylation status. We show in *in vitro* and *in vivo* in skeletal muscle in mice that antibody-mediated blockade of the β5 integrin inhibits and recombinant MFGE8 promotes PTP1B binding to and dephosphorylation of IRβ resulting in increased or reduced insulin-stimulated glucose uptake, respectively. The β5–PTP1B complex is recruited by MFGE8 to IRβ leading to termination of canonical insulin signaling. β5 blockade enhances insulin-stimulated glucose uptake in wildtype but not *Ptp1b* KO mice indicating that PTP1B functions downstream of MFGE8 in modulating insulin receptor signaling. Furthermore, in a human cohort, we report serum MFGE8 levels correlate with indices of insulin resistance. These data provide mechanistic insights into the role of MFGE8 and β5 in regulating insulin signaling.

Milk fat globule epidermal growth factor like 8 (MFGE8) is a secreted integrin ligand that regulates whole body metabolism through effects on nutrient absorption (1, 2), gastrointestinal motility (1), and lipid homeostasis (3, 4). In mice, MFGE8 impacts glucose homeostasis in multiple ways. Global *Mfge8* KO mice develop insulin resistance concomitant with the development of differences in body fat composition at 10 weeks of age (2). In contrast, we have recently reported that MFGE8 can negatively regulate insulin receptor signaling acutely (5). Antibody-mediated blockade of MFGE8 or the αvβ5 integrin, the cell surface receptor for MFGE8, leads to enhanced insulin sensitivity in wildtype (WT) mice (5). However, a detailed molecular understanding of the effect of MFGE8 and intermediaries on the insulin receptor is still lacking.

In humans, a growing body of literature links MFGE8 with insulin resistance. Serum MFGE8 levels are increased in patients with type II diabetes mellitus (T2D) and correlated with the degree of hemoglobin glycosylation (6, 7). Serum MFGE8 levels correlate with markers of insulin resistance in two diabetic cohorts of patients from China, one with gestational diabetes and one with T2D mellitus (8, 9). More recently, a missense mutation found in Punjabi Sikhs increases circulating MFGE8 and markedly increases the risk of developing T2D in this population (10, 11). These latter data are strongly suggestive of a causal relationship in humans between MFGE8 and glucose homeostasis.

Insulin signaling is tightly regulated through phosphorylation of intermediaries in the insulin receptor pathway. Insulin binding to the α subunit of the insulin receptor induces a conformational change that activates intrinsic receptor tyrosine kinase activity resulting in autophosphorylation and activation of the insulin receptor β subunit (IRβ) (12, 13). Tyrosine kinase activity of activated IRβ in turn triggers a cascade of phosphorylation events in downstream mediators ultimately inducing increased translocation of glucose transporter type 4 (GLUT-4) to the plasma membrane and increased glucose uptake (14, 15). The activity of protein tyrosine phosphatases opposes that of protein tyrosine kinases thereby dampening insulin receptor signaling (16).

As referenced above, we recently described an autoregulatory pathway through which insulin increases outer cell membrane MFGE8 levels. MFGE8 then binds the αvβ5 integrin leading to increased colocalization and binding of β5 to IRβ. This interaction promotes dephosphorylation of IRβ and reduces skeletal muscle glucose uptake in response to insulin (5). The effect of MFGE8-β5 on insulin receptor phosphorylation could be the result of reduced kinase or increased phosphatase activity. In our previous work, antibody-mediated blockade of β5 leads to delayed recovery of serum glucose levels after insulin challenge in mice (5), similar to what has been reported with genetic deletion of the phosphatase protein-tyrosine phosphatase 1B (*Ptp1b*) in mice (17). PTP1B binds to and dephosphorylates IRβ and insulin receptor substrate 1 promoting termination of the insulin signaling cascade (18, 19, 20, 21). Dysregulation of PTP1B activity has been implicated in insulin resistance and T2D (18, 22) and inhibition of PTP1B improves insulin sensitivity, glucose uptake, and glucose homeostasis in preclinical models (18, 22). In the present work, we investigate the mechanism of MFGE8-β5-mediated inhibition of insulin signaling and whether PTP1B functions downstream of MFGE8 and αvβ5 in this process. We show here that β5 is found in complex with PTP1B and that MFGE8 binding to β5 induces recruitment of this complex to IRβ thereby causing IRβ dephosphorylation and deactivation of the canonical pathway for insulin-mediated glucose uptake.

## Results

### Antibody-mediated blockade of β5 attenuates the effect of insulin on PTP1B phosphatase activity

**Figure 1:**
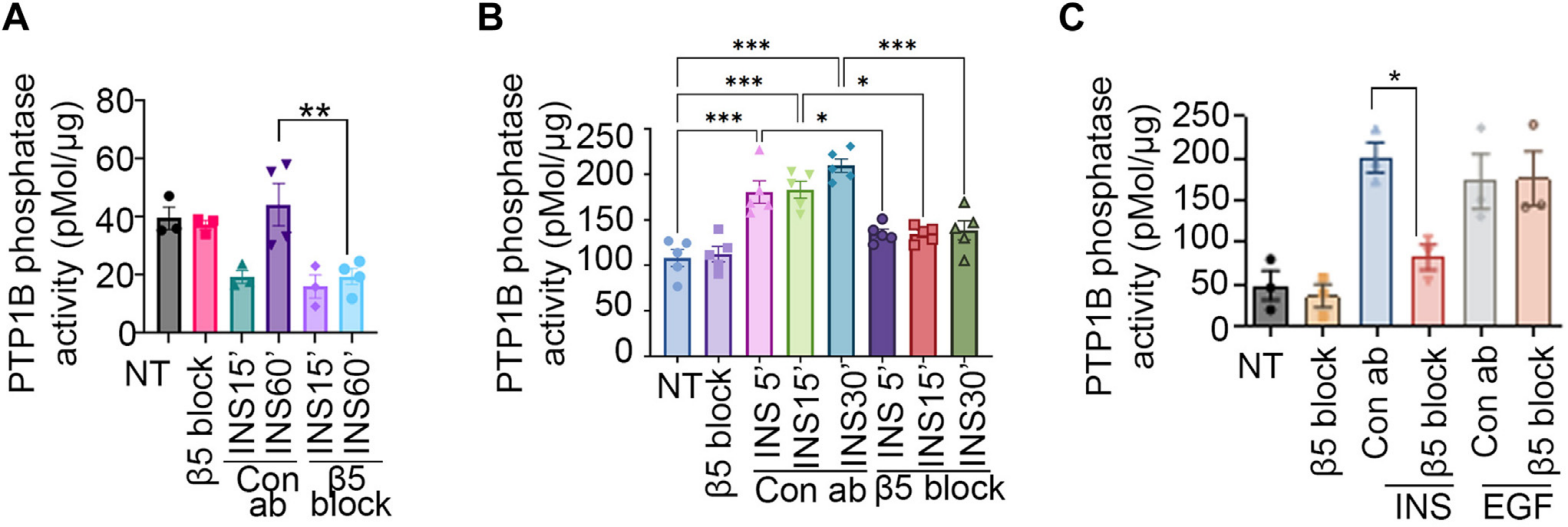
**Antibody-mediated blockade of β5 attenuates the effect of insulin on PTP1B phosphatase activity**. *A–C*, PTP1B phosphatase activity in the presence of β5 blocking or isotype control antibody in (*A*) skeletal muscle lysates harvested from WT mice 15 and 60 min after intraperitoneal insulin (1U/kg) administration, (*B*) in differentiated C2C12 myotubes 5, 15, and 30 min after insulin treatment, and (*C*) in HeLa cells 30 min after EGF or insulin (100 nm) treatment. N = 4 male mice in each group for *panel A*. Data merged from two independent experiments. N = 5 and 3 independent experiments for *panel B* and C, respectively. Data are expressed as mean ± SEM; ∗*p* < 0.05, ∗∗*p* < 0.01, ∗∗∗*p* < 0.001 and analyzed by One-way ANOVA followed by Bonferroni’s post-test. EGF, epidermal growth factor; PTP1B, protein-tyrosine phosphatase 1B.

In our recently published work (5), we showed enhanced *in vivo* skeletal muscle insulin receptor phosphorylation after insulin treatment in the presence of antibody-mediated blockade of the β5 integrin. However, the mechanism of this enhanced phosphorylation was not delineated. Therefore, we first evaluated whether antibody-mediated blockade of β5 impacts PTP1B phosphatase activity in mice. We immunoprecipitated PTP1B from skeletal muscle lysates treated with insulin in the presence of a β5 blocking or isotype control antibody and measured phosphatase activity of the immunoprecipitate on a tyrosine phosphopeptide substrate. In control samples, insulin treatment reduced PTP1B activity 15 min after administration which then recovered to baseline levels at 60 min consistent with the literature (23). In the presence of antibody-mediated blockade of β5, insulin induced a similar drop in PTP1B activity 15 min after administration. However, there was no recovery of PTP1B activity at the 60-min time point with antibody-mediated blockade of β5 (Fig. 1*A*). We also assessed the effect of antibody-mediated blockade of β5 on PTP1B phosphatase activity after insulin stimulation in differentiated C2C12 myotubes. Unlike skeletal muscle *in vivo*, insulin augmented PTP1B phosphatase activity at the time points evaluated, and this effect was inhibited by administration of β5 blocking antibody but not isotype control antibody (Fig. 1*B*). Taken together, these data show that antibody-mediated blockade of β5 attenuates the effect of insulin on PTP1B phosphatase activity.

We next evaluated whether altered PTP1B activity with antibody-mediated blockade of β5 is specific for insulin-mediated signaling. In addition to the insulin receptor, PTP1B dephosphorylates tyrosine residues of the epidermal growth factor receptor (24, 25). Therefore we used HeLa cells, which express both epidermal growth factor receptor (26) and the insulin receptor (27), to measure PTP1B phosphatase activity in the presence of β5 blocking or isotype control antibody after treatment with insulin or epidermal growth factor (EGF). Antibody-mediated blockade of β5, but not isotype control antibody, inhibited PTP1B activity stimulated by insulin but not by EGF (Fig. 1*C*) indicating that β5 specifically modulates PTP1B phosphatase activity associated with insulin signaling.

### The β5 integrin is in complex with PTP1B

**Figure 2:**
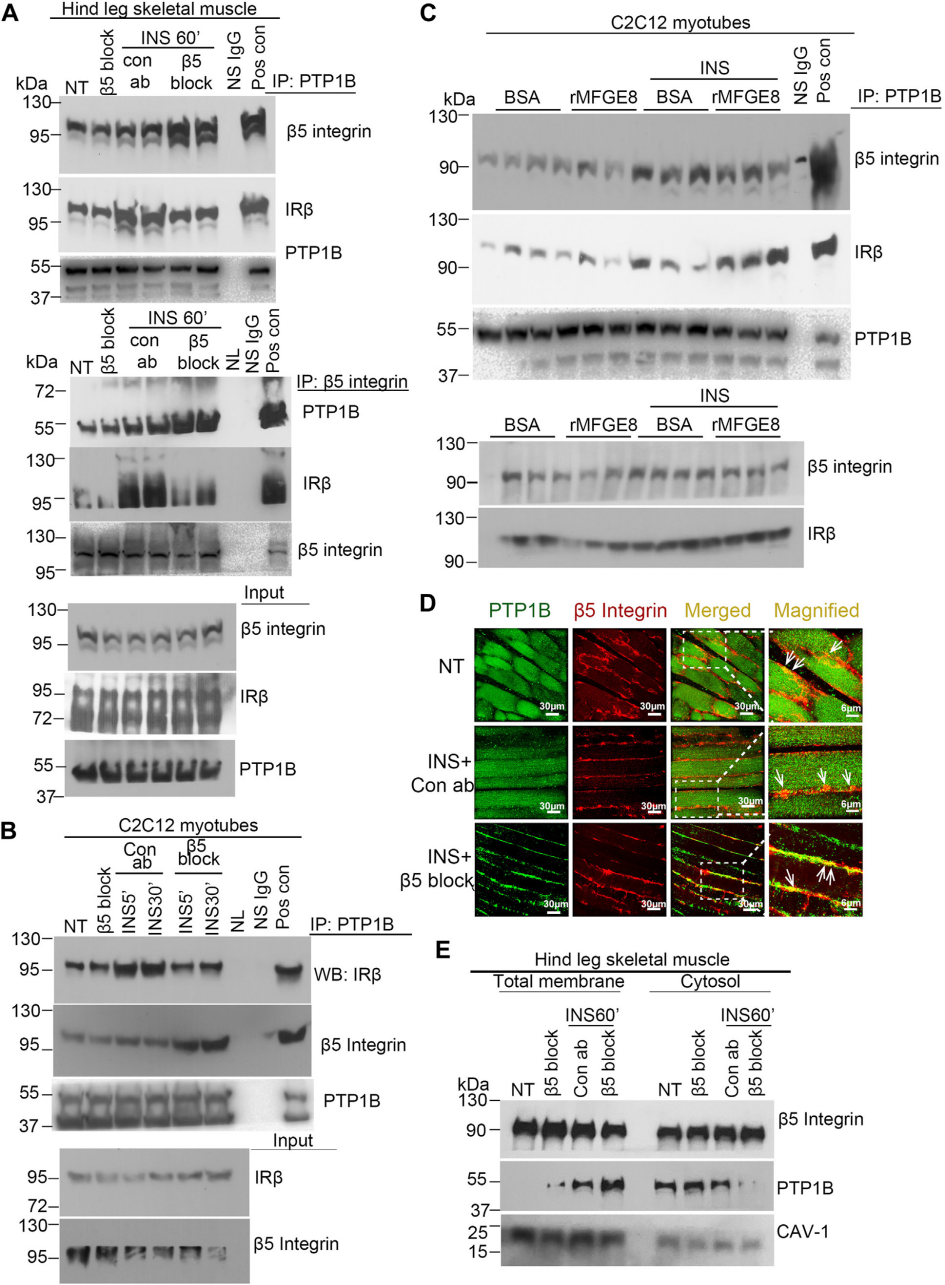
**β5 complexes with PTP1B and IRβ.***A*, co-immunoprecipitation studies showing interaction of PTP1B with β5 and IRβ in hind leg skeletal muscle lysates from mice in presence of β5 blocking or isotype control antibody 60 min after IP insulin (1U/Kg) treatment. Blots represent two independent experiments with N = 4 male mice for insulin-treated groups and two mice without insulin treatment. *B* and *C,* co-immunoprecipitation studies showing interaction of PTP1B with β5 and IRβ in C2C12 myotubes treated with (*B*) β5 blocking (5 μg/ml) or isotype control antibody or (*C*) rMFGE8 (10 μg/ml) or BSA control in the presence and absence of insulin for 30 min. N = 4 male mice in total. *Panel B* represents two independent experiments. For *panel C*, cell culture, treatment, and protein isolation performed on three different days. After immunoprecipitation, protein samples were run on the same gel for Western blot*. D,* immunostaining of PTP1B (*green*) and β5 integrin (*red*) in skeletal muscle cross sections from mice treated with insulin for 60 min in presence of β5 blocking or isotype control antibody. The *right*-most panel shows the magnified portions (delineated with the *dotted lines*) of the merged images. The *arrows* in the magnified images point to the colocalization signals (*yellow*). Images are representative from two independent experiments. *E*, cell fractionation of insulin-treated skeletal muscle tissue samples from mice pretreated with β5 blocking or isotype control antibody and insulin for 60 min, followed by Western blot for PTP1B and β5 in the cytoplasmic and membrane fraction. Western blotting for CAVEOLIN-1 (CAV-1) confirmed enrichment of the membrane fractions. N = 1 per group per experiment. Western blots are representative of three independent experiments. IP, intraperitoneal; IRβ, insulin receptor β subunit; MFGE8, milk fat globule epidermal growth factor like 8; PTP1B, protein-tyrosine phosphatase 1B; rMFGE8, recombinant MFGE8.

We next evaluated whether β5 integrin forms a complex with PTP1B *via* co-immunoprecipitation. We administered β5 blocking or isotype control antibody to WT mice 1 h before intraperitoneal (IP) insulin treatment and then performed co-immunoprecipitation studies to measure β5-PTP1B binding from hind leg skeletal muscle lysates of WT mice 1 h after insulin treatment. Lysates were immunoprecipitated with an antibody targeting PTP1B followed by Western blot for β5 or IRβ or with an antibody targeting β5 followed by Western blot for PTP1B or IRβ. Both approaches showed that β5 and PTP1B forms a complex at baseline. Insulin treatment increased co-immunoprecipitation of β5 and PTP1B which was further increased after pretreatment with the β5 blocking antibody (Fig. 2*A*). While the association between PTP1B and IRβ was increased, as expected, by insulin (28), this effect was abrogated by antibody-mediated blockade of β5 (Fig. 2*A*). These data are consistent with our previously published work showing reduced skeletal muscle insulin-stimulated interaction between β5 and IRβ in the presence of antibody-mediated blockade of β5 (5).

**Supplementary figure 1:**
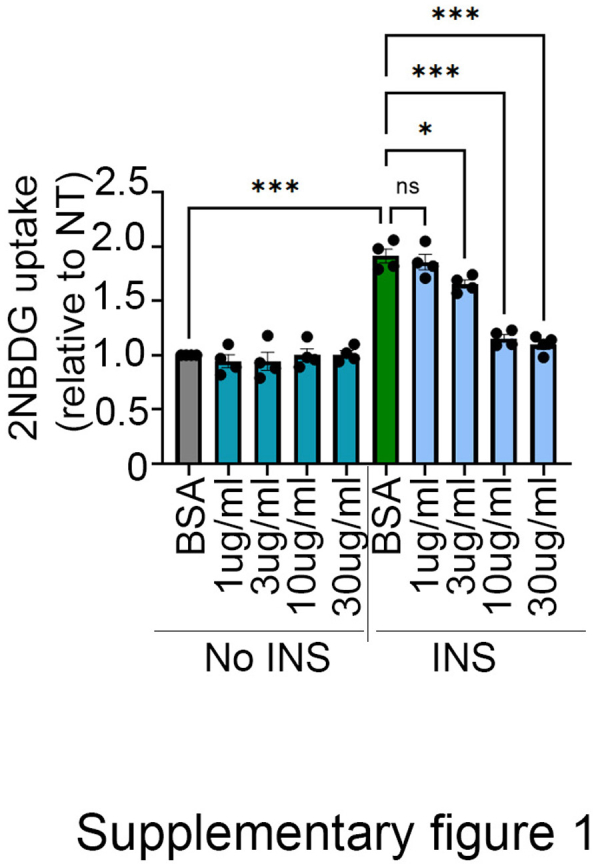
**Dose-dependent inhibition of insulin-stimulated glucose uptake by rMFGE8.** 2NBDG uptake assay in differentiated C2C12 myotubes treated with different doses of rMFGE8 (1, 3, 10 and 30μg/mL) with and without insulin for 20 minutes. N = 4 independent experiments. Data expressed as relative fold change to untreated cells (NT). Data are expressed as mean ± SEM; ∗*p* < 0.05, ∗∗∗*p* < 0.001, and analyzed by One-way ANOVA followed by Bonferroni’s post-test.

We validated these findings *in vitro* by treating differentiated C2C12 myotubes with β5 blocking or isotype control antibody in the presence of insulin for 5 and 30 min, immunoprecipitating lysates with an antibody against PTP1B, and subsequently performing Western blot for β5 and IRβ. Consistent with our *in vivo* findings (Fig. 2*A*), antibody-mediated blockade of β5 enhanced the interaction between PTP1B and β5 while reducing the interaction between PTP1B and IRβ in the presence of insulin at both time points (Fig. 2*B*). Of note, differentiated C2C12 myotubes had dose-dependent inhibition of insulin-stimulated glucose uptake by recombinant MFGE8 (rMFGE8) (Fig. S1) These data show that PTP1B complexes with β5 integrin, consistent with the functional interaction suggested by the data in Fig. 1.

We have previously shown that the effect of β5 integrin on insulin signaling is specific for MFGE8 and not induced by other β5 ligands such as fibronectin or vitronectin (5). To determine whether the β5–PTP1B interaction is modified by MFGE8, we treated differentiated C2C12 myotubes with rMFGE8 with and without insulin for 30 min and performed co-immunoprecipitation of cell lysates with an antibody targeting PTP1B followed by Western blot for β5 and IRβ. rMFGE8 treatment did not impact the association between β5 and PTP1B at baseline or after insulin treatment (Fig. 2*C*). However, in the presence of insulin, rMFGE8 increased the interaction between IRβ and PTP1B (Fig. 2*C*). Taken together, these data indicate that MFGE8 increases recruitment of the β5–PTP1B complex to IRβ.

We next performed immunofluorescence staining for β5 and PTP1B in sections from hind leg skeletal muscle of mice treated with insulin for 60 min in the presence of either β5 blocking or isotype control antibody. We found that β5 and PTP1B colocalized (Fig. 2*D*) and that colocalization increased after insulin treatment in the presence of β5 blocking antibody consistent with our co-immunoprecipitation experiments (Fig. 2*D*). Interestingly, there was a striking enrichment of membrane localization of PTP1B after insulin treatment with antibody-mediated blockade of β5 (Fig. 2*D*).

To further investigate the subcellular localization of PTP1B and β5 integrin, we performed cell fractionation of skeletal muscle lysates harvested from the hind legs of WT mice 60 min after insulin treatment and pretreated with β5 blocking or isotype control antibody. Consistent with our immunofluorescence data, antibody-mediated blockade of β5 led to increased membrane and reduced cytosolic PTP1B expression after insulin treatment (Fig. 2*E*). Taken together, these data suggest that by preventing MFGE8 binding to β5, antibody-mediated blockade of β5 leads to sequestration of PTP1B in complex with the integrin at the cell membrane; this sequestration subsequently reduces recruitment of the complex to IRβ and functionally decreases PTP1B-mediated dephosphorylation of IRβ.

### Antibody-mediated blockade of β5 promotes insulin-stimulated AKT S473 phosphorylation

**Figure 3:**
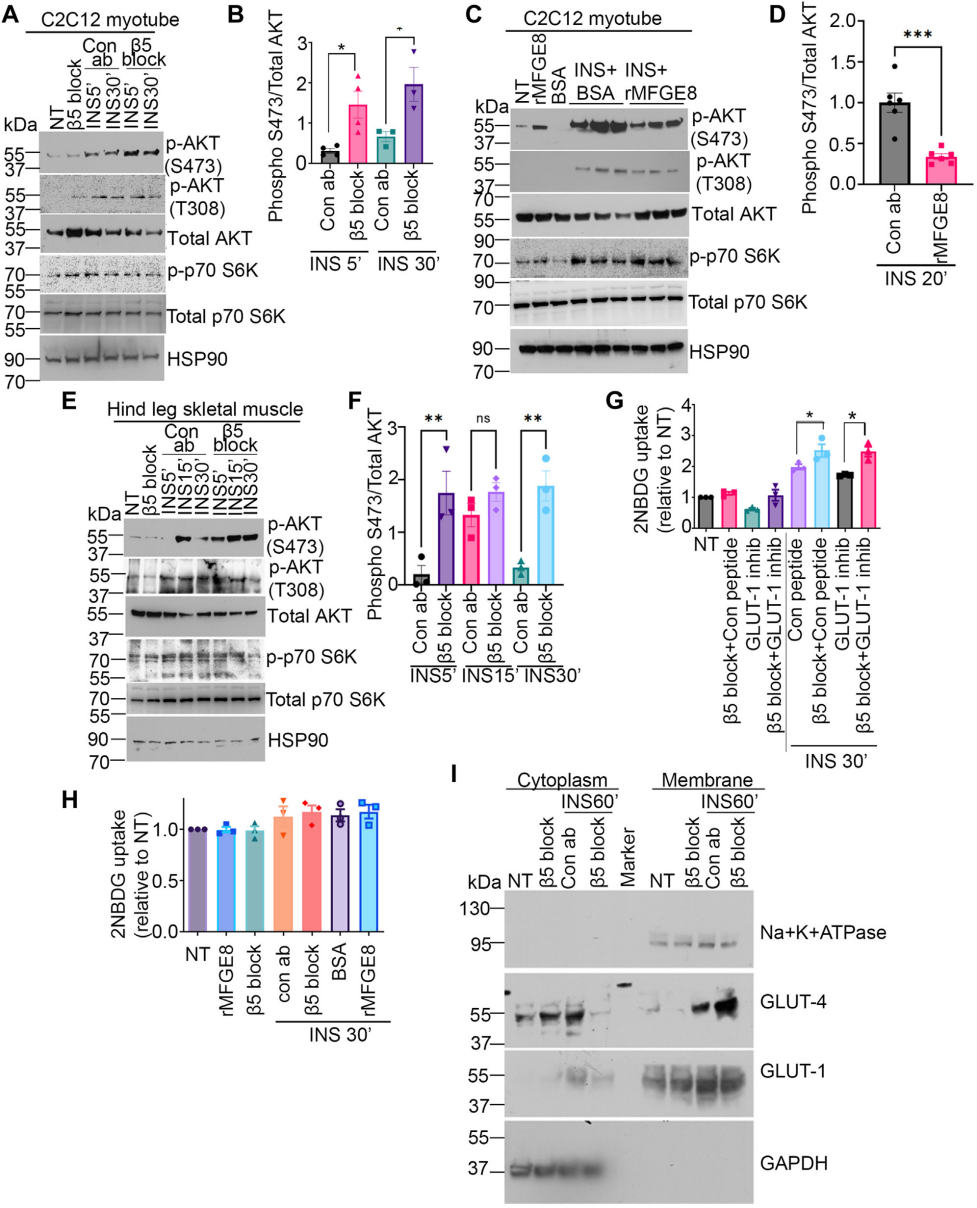
**Antibody-mediated blockade of β5 activates canonical insulin signaling**. *A,* representative Western blot showing phosphorylated (serine 473 residue, S473; Threonine 308 residue, T308) AKT, total AKT, phosphorylated p70 S6Kinase, and total p70 S6K levels in differentiated C2C12 myotubes in the presence of β5 blocking or isotype control antibody treated with insulin for 30 min. N = 3 independent experiments. *B*, densitometric analysis of p-AKT (S473) Western blots including *panel A*. *C–D,* representative Western blot showing p-AKT (S473), p-AKT(T308), total AKT, p-p70S6K, and total p70S6K levels in differentiated C2C12 myotubes treated with rMFGE8 (10 μg/ml) and insulin for 20 min. N=3 to 6 independent samples from 1 to 2 independent experiments. *D*, densitometric analysis of Western blot of p-AKT (S473) including *panel C*. *E*, representative Western blot of phosphorylated and total AKT levels in skeletal muscle lysates from mice treated with insulin for 5, 15, and 30 min in presence of β5 blocking or isotype control antibody. The Western blot shown is representative of three independent experiments. *F*, densitometric analysis of Western blot of p-AKT (S473) including *panel E*. *G*, effect of GLUT-1 inhibitor (1 μM) or control peptide on 2NBDG uptake in C2C12 myotubes treated with β5 blocking or isotype control antibody after insulin stimulation. N = 3 independent experiments. Data expressed as relative fold change to untreated cells (NT). *H,* effect of antibody-mediated blockade of β5 (5 μg/ml) or rMFGE8 treatment (10 μg/ml) on 2NBDG uptake in 3T3 fibroblasts in the presence or absence of insulin. N = 3 independent experiments. *I*, Western blot showing GLUT-1 and GLUT-4 levels in cytosolic and membrane fractions isolated from C2C12 myotubes treated with insulin for 30 min in presence of β5 blocking or isotype control antibody. Na^+^K^+^ATPase and GAPDH were used as loading controls for membrane and cytosolic fractions, respectively. The Western blot represents three independent experiments. Data are expressed as mean ± SEM; ∗*p* < 0.05, ∗∗*p* < 0.01, ∗∗∗*p* < 0.001, and analyzed by One-way ANOVA followed by Bonferroni’s post-test. GLUT-1, glucose transporter 1; GLUT-4, glucose transporter type 4; MFGE8, milk fat globule epidermal growth factor like 8; rMFGE8, recombinant MFGE8.

**Supplementary figure 2:**
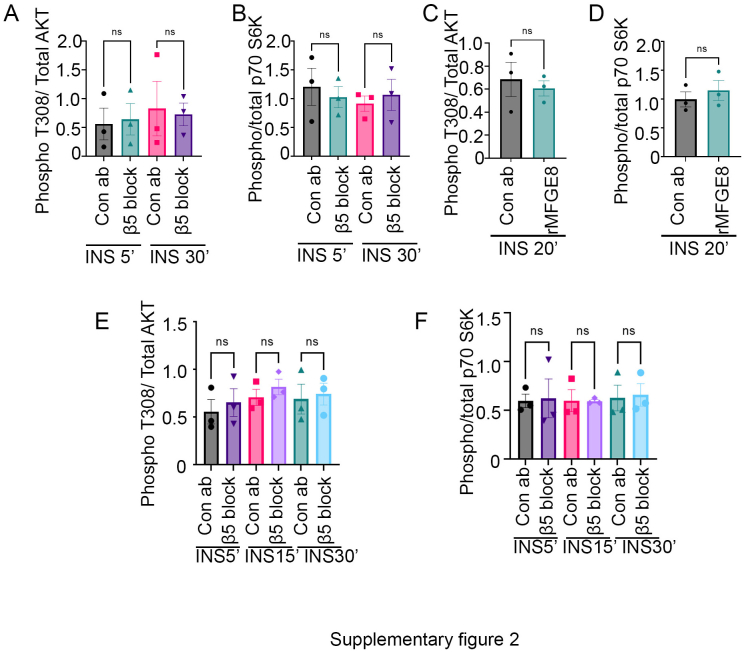
**Densitometric analysis showing no effect of MFGE8/ β5 signaling on phosphorylation of AKT (T308) and p70S6 Kinase.***A* and *B*, Densitometric analysis of western blots including figure 3A for (*A*) phosphorylated AKT (Threonine 308 residue, T308) and (*B*) phosphorylated p70 S6Kinase in differentiated C2C12 myotubes in the presence of β5 blocking or isotype control antibody treated with insulin for 30 minutes. N = 3 independent experiments. *C* and *D*, Densitometric analysis of western blots in Figure 3*B* showing (*C*) phosphorylated AKT T308 and (*D*) phosphorylated p70 S6Kinase levels in differentiated C2C12 myotubes in the presence of rMFGE8 and insulin. N = 3 independent samples. *E* and *F*, Densitometric analysis of western blots including Figure 3*C* showing (*E*) phosphorylated AKT T308 and (*F*) phosphorylated p70S6K levels in skeletal muscle lysates from mice treated with insulin for 5, 15 and 30 minutes in presence of β5 blocking or isotype control antibody. N = 3 independent experiments. Data are expressed as mean ± SEM; and analyzed by One-way ANOVA followed by Bonferroni’s post-test.

We have previously shown that MFGE8/β5 impacts insulin-mediated glucose uptake *via* the PI3 kinase (PI3K) pathway (5). Insulin-stimulated activation of PI3K signaling affects the mTORC1, mTORC2, and PDK1 signaling pathways. While mTORC1 and mTORC2 mediate Akt phosphorylation at serine 473 residue (S473) and p70 S6K1 phosphorylation at threonine 389 residue (T389), respectively, PDK1-mediates Akt phosphorylation at threonine 308 (T308). To determine how MFGE8 and β5 impact canonical insulin signaling, we pretreated differentiated C2C12 myotubes with β5 blocking antibody for 1 h, followed by insulin for 5 or 30 min, and evaluated AKT S473, AKT T308, and p70 S6K1 T389 phosphorylation by Western blot. Antibody-mediated blockade of β5 augmented insulin-stimulated AKT S473 phosphorylation at both timepoints (Fig. 3, *A* and *B*) with minimal effects on AKT T308 and p70 S6K1 T389 phosphorylation (Figs. 3*A* and S2, *A* and *B*). Additionally, treatment with rMFGE8 dampened insulin-mediated AKT S473 phosphorylation without impacting AKT T308 and p70 S6K1 T389 phosphorylation (Figs. 3, *C* and *D* and S2, *C* and *D*). We interpret these data to suggest that the MFGE8-β5 pathway predominantly impacts mTORC2-dependent signaling downstream of PI3K. To validate these findings *in vivo*, we administered β5 blocking or isotype control antibody to WT mice 1 h before treating them with insulin (5, 15, or 30 min) and assessed AKT S473 phosphorylation in hind leg skeletal muscle protein lysates. Antibody-mediated blockade of β5 enhanced AKT S473 phosphorylation at the 5- and 30-min timepoints as compared with control antibody-treated mice (Figs. 3, *E* and *F* and S2, *E* and *F*). We interpret these data to indicate that antibody-mediated blockade of β5 induces enhanced and persistent activation of canonical insulin signaling pathway.

### Antibody-mediated blockade of β5 increases glucose uptake through GLUT-4

Next, we evaluated how antibody-mediated blockade of β5 impacts glucose transporter activity. Skeletal muscle predominantly utilize glucose transporter 1 (GLUT-1) and GLUT-4 for glucose uptake (14, 29, 30). We treated differentiated C2C12 myotubes with a small molecule inhibitor of GLUT-1 in the presence of β5 blocking or isotype control antibody. The ability of antibody-mediated blockade of β5 to augment insulin-mediated glucose uptake in these cells was unaffected by GLUT-1 inhibition (Fig. 3*G*). We subsequently evaluated whether β5 blockade impacts insulin-stimulated glucose uptake in 3T3 fibroblasts, a cell line that expresses GLUT-1 (31) but not GLUT-4 (32). β5 blockade had no effect on insulin-stimulated glucose uptake in these cells suggesting that the effect of β5 is independent of GLUT-1 (Fig. 3*H*). We next performed cell fractionation and assessed cytoplasmic and membrane levels of GLUT-1 and GLUT-4 in insulin-treated differentiated C2C12 myotubes in the presence of β5 blocking or isotype control antibody. Antibody-mediated blockade of β5 markedly increased membrane enrichment of GLUT-4 without an appreciable effect of membrane GLUT-1 expression in the presence of insulin (Fig. 3*I*). Taken together, these data indicate that antibody-mediated blockade of β5 impacts insulin-stimulated glucose uptake in myotubes by promoting GLUT-4 movement to the cell membrane.

### β5 dampens insulin signaling *via* PTP1B

**Figure 4:**
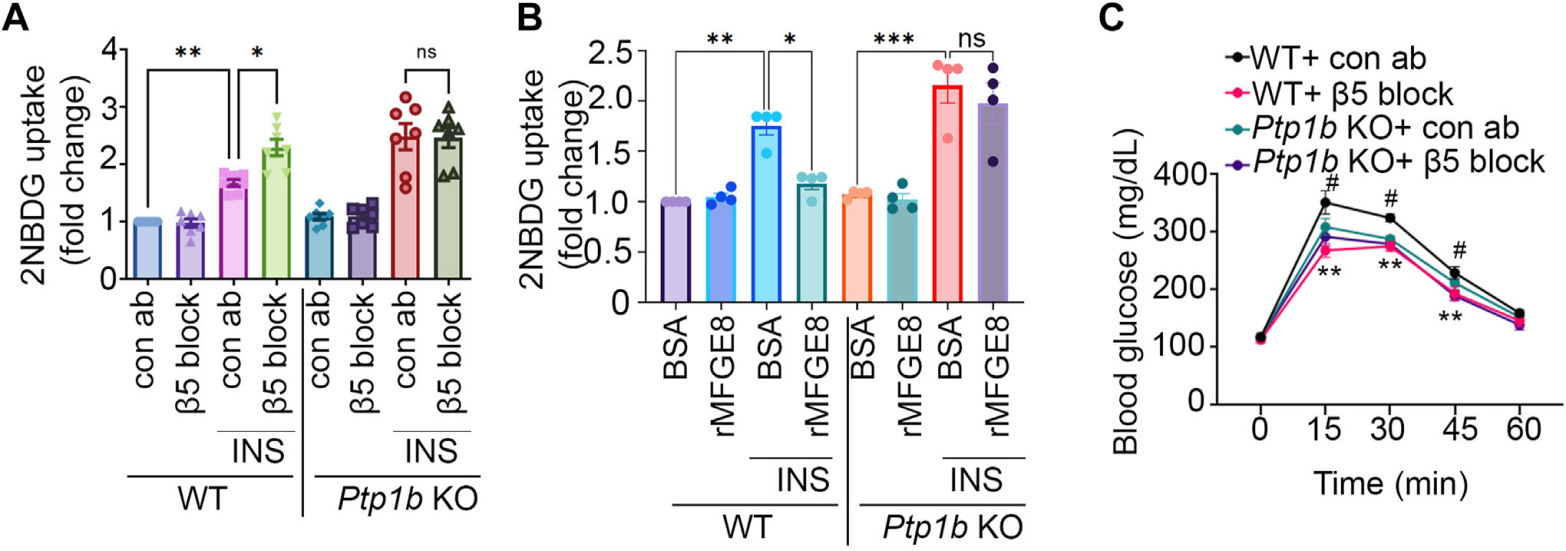
**Antibody-mediated blockade of β5 impacts insulin signaling through PTP1B**. *A* and *B,* 2NBDG uptake assay in WT and *Ptp1b* KO myotubes in presence of (*A*) β5 blocking or isotype control antibody or (*B*) rMFGE8 or BSA with and without insulin treatment. N = 6 independent experiments for *panel A*, and N = 4 independent experiments for *panel B*. *C*, glucose tolerance test in 7- to 8-week-old male WT and *Ptp1b* KO mice after intraperitoneal injection of β5 blocking or isotype control antibody. N = 6 male mice per group from two independent experiments are presented. Data are expressed as mean ± SEM. Data in panel (*A* and *B*) were analyzed by one-way ANOVA followed by Bonferroni’s post-test. ∗*p* < 0.05, ∗∗*p* < 0.01 and ∗∗∗*p* < 0.001. Data in panel (*C*) were analyzed by 2-way ANOVA followed by Tukey’s post-test. ∗∗*p* < 0.01, ∗*p* < 0.05, when comparing WT+Con ab *versus* WT+ antibody-mediated blockade of β5 (β5 block) groups; #*p* < 0.05 when comparing WT+Con ab *versus Ptp1b*KO+Con ab groups. MFGE8, milk fat globule epidermal growth factor like 8; PTP1B, protein-tyrosine phosphatase 1B; rMFGE8, recombinant MFGE8.

**Figure 5:**
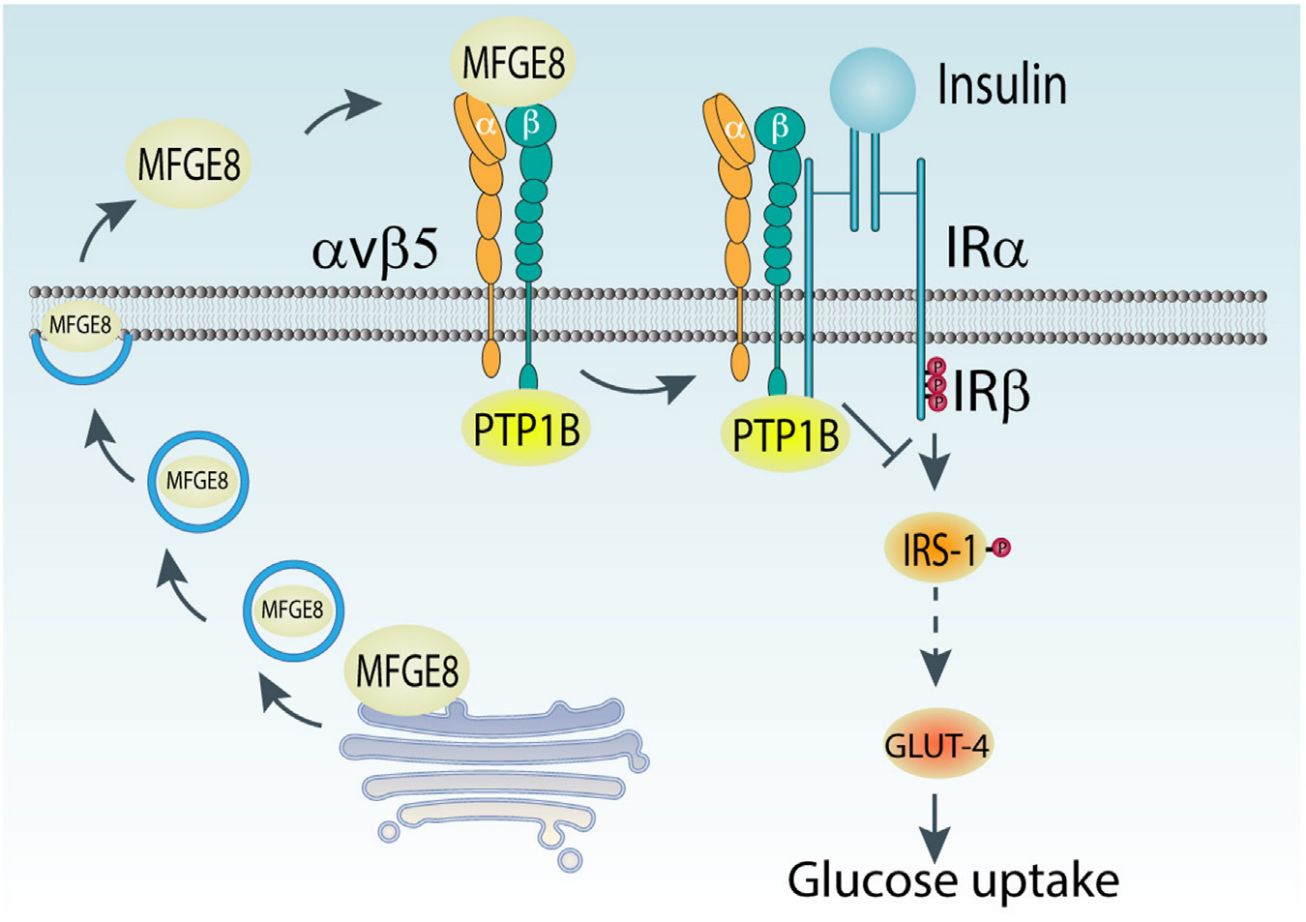
**Model depicting how MFGE8 impacts insulin signaling through PTP1B**. MFGE8 binding of the β5 integrin on the outer cell membrane leads to recruitment of the β5-PTP1B complexes to IRβ. PTP1B subsequently dephosphorylates IRβ leading to reduced translocation of GLUT4 to the cell surface and reduced glucose uptake in response to insulin. GLUT-4, glucose transporter type 4; IRβ, insulin receptor β subunit; MFGE8, milk fat globule epidermal growth factor like 8; PTP1B, protein-tyrosine phosphatase 1B.

To determine whether PTP1B is necessary for the effect of β5 on insulin signaling, we measured insulin-mediated glucose uptake in *Ptp1b* KO myotubes after treating cells with β5 blocking or isotope control antibody. *Ptp1b* KO myotubes showed enhanced insulin-mediated glucose uptake compared to WT myotubes that was not affected by antibody-mediated blockade of β5 (Fig. 4*A*). Furthermore, while rMFGE8 treatment dampened insulin-mediated glucose uptake in WT myotubes, it had no effect in *Ptp1b* KO myotubes indicating that MFGE8 inhibits insulin signaling through PTP1B (Fig. 4*B*). To validate our findings *in vivo*, we administered β5 blocking or isotype control antibody to WT and *Ptp1b* KO mice and performed a glucose tolerance test. *Ptp1b* KO mice had significantly reduced blood glucose levels after IP glucose challenge as compared with WT mice. Antibody-mediated blockade of β5 in *Ptp1b* KO mice did not further impact blood glucose level (Fig. 4*C*). Taken together, these data show no additive effect on insulin-stimulated skeletal muscle glucose uptake with simultaneous silencing of *Ptp1b* and *β5* and support the hypothesis that PTP1B functions downstream of MFGE8 and β5 in modulating insulin signaling (Fig. 5) (5).

### Physiological regulation of PTP1B/β5 interaction

**Figure 6:**
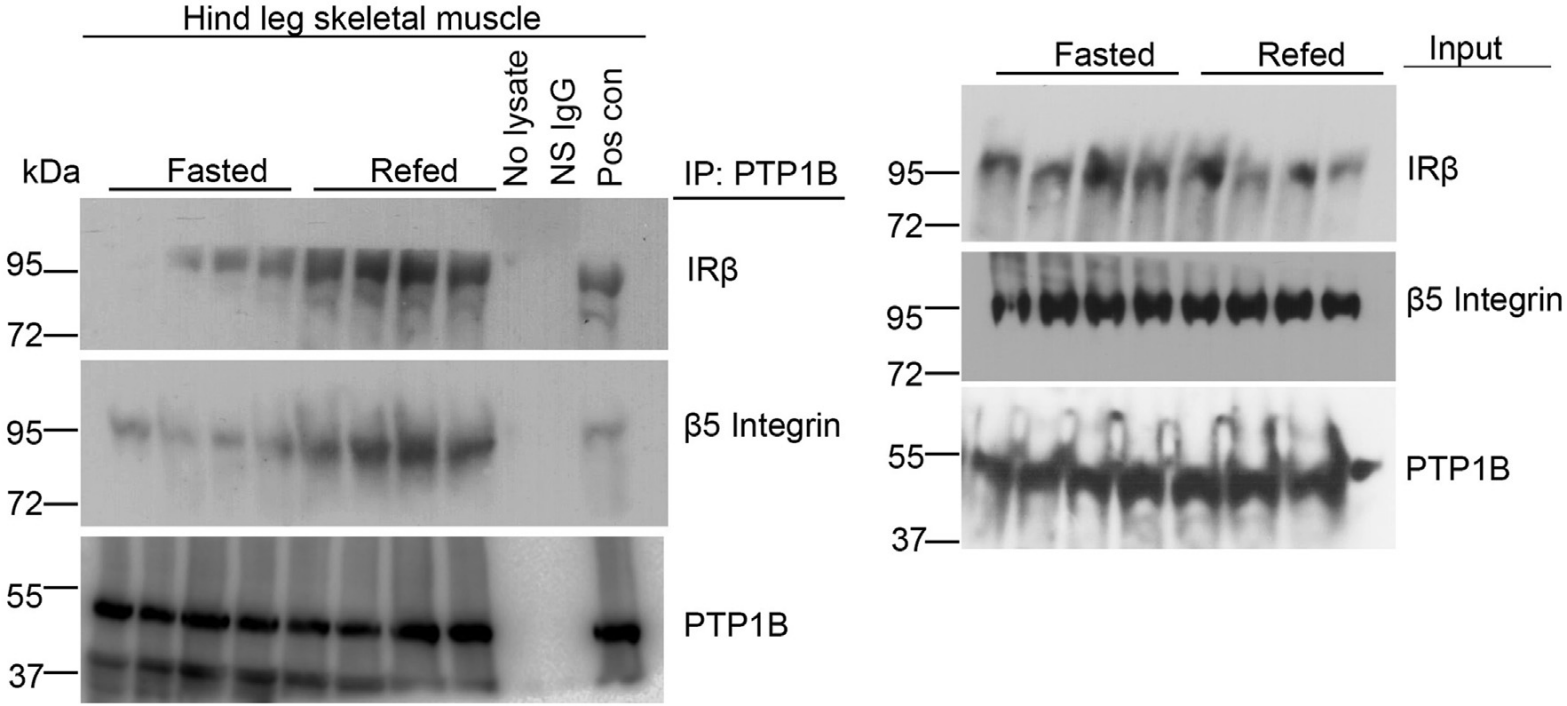
**Physiological regulation of PTP1B/β5 interaction**. Co-immunoprecipitation studies showing interaction of PTP1B with β5 and IRβ in hind leg skeletal muscle lysates from mice fasted for 16 h or mice refed for 1 h after a 16 h fast. N = 4 mice in each group. IRβ, insulin receptor β subunit; PTP1B, protein-tyrosine phosphatase 1B.

We have previously shown that fasting decreases while refeeding increases serum MFGE8 and insulin levels and the association of IRβ and β5 in skeletal muscle (5). To examine whether refeeding increases the association of IRβ and PTP1B, we immunoprecipitated PTP1B from skeletal muscle lysates from fasted and refed mice and performed Western blot for IRβ and β5. Refeeding increased the association between PTP1B and IRβ as well as interactions between PTP1B and β5 as compared with the fasted state (Fig. 6) indicating that these interactions are regulated by nutritional status and further highlighting the role of this pathway under physiological conditions.

### Correlation between serum Mfge8 and insulin resistance in human subjects

Serum MFGE8 levels are correlated with indices of insulin resistance in Chinese cohorts of patients with T2D (8) or gestational diabetes (9). To expand on these findings, we examined serum MFGE8 levels and indices of insulin resistance in a multi-ethnic cohort of 25- to 65-year-old healthy and primarily White, Chinese, and Hispanic individuals living in the San Francisco Bay Area (Table S1). We performed multivariate linear regression analysis utilizing significant independent predictor variables determined by Spearman correlation coefficients (Table S2) to identify independent predictors of insulin resistance as measured by the homeostatic model assessment of insulin resistance (HOMA-IR). MFGE8 was a significant independent predictor of insulin resistance in this cohort on par with what we found for blood glucose, fasting glucose, and insulin levels (Table S3).

### Discussion

Despite many decades of work, there is still much unknown about insulin signaling and glucose homeostasis. The regulation of metabolic homeostasis is enormously complex, but elucidating this complexity can yield insights not just about normal physiology but also about disease states. The ongoing global pandemic of obesity and one of its key complications, T2D, highlight the importance of understanding all pathways regulating glucose and insulin signaling.

We recently identified an autoregulatory pathway by which insulin promotes termination of insulin receptor signaling by increasing the amount of MFGE8 at the outer cell membrane (5). MFGE8 binds the αvβ5 integrin, increasing the association of αvβ5 with the insulin receptor and resulting in insulin resistance through reduced phosphorylation/activation of the insulin receptor signaling pathway (5). In the current work, we build on these findings and show that decreased insulin receptor phosphorylation induced by MFGE8 and αvβ5 occurs *via* the phosphatase PTP1B. Our data support a model wherein β5, PTP1B, and IRβ are in a complex that is modulated by MFGE8 and β5. The interaction of MFGE8 with β5 increases the association between PTP1B and IRβ. By contrast, our co-immunoprecipitation studies indicate that blockade of the integrin in the presence of insulin increases the association of β5 with PTP1B while decreasing the association with IRβ. Our immunofluorescence and cell fractionation studies validate these findings by showing increased membrane localization of PTP1B in the presence of insulin and integrin blockade suggesting that ligand (MFGE8) binding to β5 releases the β5–PTP1B complex from the membrane such that it can strengthen its association with IRβ. PTP1B is known to be important in insulin receptor desensitization (19), increased PTP1B expression is associated with insulin resistance (17, 33) and PTP1B is being evaluated as a therapeutic for patients with T2D (34). Our data add to the field by demonstrating a mechanism that regulates PTP1B activity during insulin signaling. Furthermore, our data suggest that biological manipulation of β5 may provide an alternate or additional mechanism to inhibit PTP1B that could be of therapeutic value.

There is an increasing body of literature in human subjects supporting the role of MFGE8 in diabetes mellitus. From our perspective, the most intriguing data come from work showing that a missense mutation in MFGE8 dramatically increases the risk for developing T2D in Punjabi Sikhs, a population prone to developing nonobese T2D (10). Additional studies of interest include two cohorts of patients from China, one with T2D, and one with gestational diabetes in whom serum MFGE8 levels correlate with multiple indices of insulin resistance (8, 9). Interestingly, in the T2D cohort, the association is prominent in nonobese subjects and absent in obese subjects (8). We were therefore curious to examine this relationship in a North American cohort consisting of similar proportion of healthy White, Chinese, and Hispanic individuals from the general population. Our findings are consistent with the previously published correlation of serum MFGE8 levels with indices of insulin resistance (8, 9). In our cohort, serum MFGE8 levels significantly predict HOMA-IR on par with what we observed for fasting glucose and insulin levels. Interestingly, this correlation was not restricted to nonobese patients in contrast to what is been reported in the Chinese cohort (8). Despite relatively modest sample sizes, these four cohorts (8, 9, 10) validate our mechanistic work in mice indicating a role for MFGE8 in inducing insulin resistance (2, 5) and lay the foundation for therapeutic targeting of this pathway to ameliorate insulin resistance.

There still remain a number of unanswered questions in our work regarding the interplay between MFGE8, insulin, and insulin signaling under physiological conditions. Insulin has been shown to induce Mfge8 production *in vitro* (35). We have previously demonstrated that insulin coordinates inhibition of its own signaling pathway by increasing the amount of MFGE8 at the outer cell membrane where it can bind to the αvβ5 integrin (5). This type of effect coupled with the relatively low serum levels of MFGE8 as compared with tissue levels make it somewhat difficult to understand the actual physiological concentrations of MFGE8 necessary for a particular function *in vivo* (2). The signals that control production and secretion of MFGE8 also remain incompletely described and are an active focus of our laboratory, in particular as it relates to downstream effectors of insulin-induced MFGE8 production. The cellular source for the increased serum levels of MFGE8 in patients with T2D is an interesting question that is challenging to experimentally evaluate given numerous cell types produce MFGE8 (1, 4, 5, 36, 37) and that, as a secreted molecule, MFGE8 can be made by one cell type and passed to an adjacent population that uses, but does not produce, MFGE8 (38). Additionally, circulating MFGE8 could be produced by immune cells in the blood though it seems more logical that it is made in organs and secreted into the circulation. The relatively ubiquitous expression pattern and soluble nature of MFGE8 make a study of this protein less amenable to traditional tissue-specific gene deletion strategies. The effect of obesity on the insulin-MFGE8-β5-PTP1B pathway we have described remains to be determined. Interestingly, however, the increased incidence of T2D in Punjabi Sikhs with the missense mutation in MFGE8 strongly suggests in humans that the effect of MFGE8 on insulin signaling is clinically relevant in nonobese T2D patients (11). We can conclude from our previous work examining circulating blood glucose, insulin, and MFGE8 levels in the fasting and refed state (5) and our current work looking at the association between PTP1B and IRB in the fasting and refed state (Fig. 6) that the MFGE8-β5 pathway, under physiological conditions, is regulated by nutritional status. Furthermore, insulin coordinating inhibition of insulin signaling through MFGE8 provides a physiological feedback mechanism to prevent the possibility of persistent and/or excessive activation of insulin receptor signaling which can lead to life-threatening hypoglycemia.

One limitation of our work is that we cannot rule out the potential impact of antibody-mediated blockade of β5 on the intrinsic tyrosine kinase activity of the insulin receptor independent of its effect on PTP1B. We have also not explored whether increasing the association of β5 with IRβ impacts insulin receptor structure such that it is less receptive to insulin binding thereby reducing the strength of insulin signaling.

A better understanding of pathobiology and etiology of insulin resistance can open the door to new therapeutic avenues to treat patients. Although insulin therapy is the mainstay of treatment for type 1 diabetes mellitus and is a major component of therapeutic regimes for patients with T2D, chronic insulin treatment can have numerous deleterious effects. For example, insulin can promote weight gain, a particularly disadvantageous effect for obese patients with T2D. Insulin therapy in insulin resistant patients can require the administration of very high doses of exogenous insulin raising the risk of serious consequences from dosing errors. Therefore approaches that leverage endogenous pathways to enhance insulin signaling could be one means to avoid some of these problems. Therefore, we believe our work is an important addition to understanding foundational mechanisms of insulin signaling regulation.

## Experimental procedures

### Mice

All studies utilized 6- to 10-week-old male age-matched mice in C57/bl6 background. *Ptp1b* KO mice are in the C57/bl6 background and have been extensively characterized (17, 39).

### Cell culture and treatments

C2C12 myoblasts (ATCC) were cultured in Dulbecco's modified Eagle's medium supplemented with 10% FBS at 37 °C and 5% CO_2_. Cells were then passaged and differentiated following previously published protocol (5). The experiments were performed after 4 days of differentiation. C2C12 cells were serum-starved for 4 h. Treatment with β5 blocking (5 μg/ml) antibody or a control antibody was administered 1 h before insulin (100 nm) stimulation. rMFGE8 (10 μg/ml) or BSA control was added to the media concurrently with insulin.

HeLa cells were cultured in Eagle’s minimal essential medium supplemented with 10% FBS at 37 °C and 5% CO_2._ HeLa cells were treated with 100 nm insulin and EGF for 30 min β5 blocking (5 μg/ml) antibody, or a control antibody was administered 1 h before insulin or EGF treatment.

### Primary myoblast isolation

Primary myoblast from Hind leg skeletal muscles of WT and Ptp1b KO mice were isolated following previously published protocol (5). Cells were cultured on matrigel precoated dish at 37 °C CO_2_ incubator and passaged when at 70% confluence. Cells were preplated on nonmatrigel-coated dishes for 1 h after each passage to remove nonmyoblast population. To differentiate myoblasts into myotubes, myoblasts were cultured to 90% confluency and subsequently cultured in differentiation media (2% horse serum and 1% Pen-Strep in Dulbecco's modified Eagle's medium) for 3 to 4 days.

### Protein isolation and Western blot

For protein isolation, cells were scraped in PBS, washed, and centrifuged. Proteins from cell pellets were isolated using protein isolation buffer (20 mM Tris-HCl pH8.0, 137 mM NaCl, 1% Nonidet P-40, and 2 mM EDTA). To isolate protein from hind leg skeletal muscles, vastus and gastrocnemius muscle tissues were dissected, chopped into pieces, and washed in cold PBS two times. Tissues were then lysed in protein isolation buffer using a tissue-lyser (Qiagen). Protein concentration was measured by Bradford assay. Protein samples (20 to 40 μg) were resolved by SDS-PAGE in 7.5% gels (Bio-Rad) and transblotted onto polyvinylidene fluoride membranes (Millipore). Membranes were blocked with 5% BSA-TBST for 1 h and then incubated with primary antibody overnight at 4 ˚C. Membranes were incubated with HRP-conjugated secondary antibodies for 1 h and washed, and bands were generated using an Immobilon Western chemiluminescence HRP-conjugated substrate (Amersham) and developed on a film (Kodak). Membranes were deprobed using Restore Western blot stripping buffer (Thermo scientific) and reprobed for other primary antibodies.

### Co-immunoprecipitation

Proteins were isolated in nondenaturing lysis buffer (20 mM Tris-HCl pH8.0, 137 mM NaCl, 1% Nonidet P-40, and 2 mM EDTA) and then precleared using fast-flow protein G Dyna beads. Precleared proteins (200 to 300 μg) were incubated overnight with the primary antibodies against PTP1B or β5 integrin or a nonspecific IgG control antibody. The immunoprotein complex was then attached to protein G Dyna beads followed by protein elution from the beads in 1% SDS buffer by heating at 60 ˚C. Equal volumes of the eluates were used for immunoblotting using antibodies against IRβ, β5 integrin, and PTP1B. Proteins immunoprecipitated using nonspecific IgG were negative control for specific antibodies. Immunoblotting with total protein lysates was used as input samples. One of the input samples was run on the same gel with the immunoprecipitated proteins to use it as a positive control.

### PTP1B phosphatase activity assay

PTP1B phosphatase activity assay was performed using a tyrosine phosphatase assay system (Promega). Endogenous phosphates were removed from the tissue extracts or cell lysates using sephadex spin column which were subsequently incubated and immunoprecipitated with an antibody targeting PTP1BA reaction mix was then prepared using a tyrosine phosphopeptide substrate (ENDp(Y)INASL). Immunoprecipitated protein (2 μg) was incubated with the substrate for 20 min at 37 °C in a 96-well plate. The reaction was terminated using molybdate dye/additive mixture for 15 min at room temperature. Absorbance of the samples was measured using a plate reader at 630 nm. Phosphatase activity was calculated using the phosphatase assay standard supplied with the kit.

### Glucose uptake assay

The 2NDBG uptake assay in C2C12 myotubes and mouse primary skeletal myotubes was performed using a commercial assay kit (Cayman chemicals) following previously published protocol (5). Cells were preincubated with insulin (100 nM) for 10 min followed by the addition of nonhydrolyzable fluorescent glucose analog 2NBDG (10 mg/ml) for 30 min in the same media. Fluorescent intensity of cellular 2NBDG (excitation: 488 nm and emission: 535 nm) was measured using a plate reader. Fluorescent intensities of 2NBDG were then normalized to wheat germ agglutinin (WGA 680) staining intensities.

### Glucose tolerance test

Six- to eight-week-old male WT and *Ptp1b* KO mice were fasted for 5 h and then injected IP with 2 gm/kg glucose. Mice were treated with IP β5 blocking antibody (5 mg/kg) (clone ALULA, provided by A. Atakilit, University of California San Francisco [UCSF]) or mouse isotype control antibody (clone 2H6-C2, ATCC CRL-1853) for 1 h before glucose administration.

### *In vivo* treatment with β5 blocking antibody

Mice were fasted for 4 h before injecting them IP with β5 blocking (5 mg/Kg) or isotype control antibody for 1 h. Mice were then treated with IP insulin (1 U/kg) for 15 or 60 min before harvesting the hind leg skeletal muscle tissues (5).

### Fasting and refeeding of mice

Mice were fasted for 16 h followed by refeeding with normal chow diet for 1 h prior to obtaining skeletal muscle tissue samples. Mice had free access to the food during the refeeding period.

### Human subjects

All subjects signed consent forms to participate in the study, which was approved by the UCSF Institutional Review Board. Subjects were part of a multiethnic clinical cohort, termed the Inflammation, Diabetes, Ethnicity, and Obesity, consisting of 25- to 65-year-old healthy men and women living in the San Francisco Bay Area and recruited from medical and surgical clinics at the UCSF and the Zuckerberg San Francisco General Hospital or through local public advertisements. The subjects covered a wide range of body mass index (18.5–52 kg/m2). Individuals were excluded for smoking, not being weight-stable for the last 3 months (change >3%), having any acute or chronic inflammatory or infectious disease, liver failure, renal dysfunction, cancer, or alcohol consumption >20g per day. Inflammation, Diabetes, Ethnicity, and Obesity collects demographic, medical, dietary, and lifestyle data from all subjects using validated questionnaires. Human studies reported in this manuscript abide by the Declaration of Helsinki principles.

### Anthropometric and body composition measurements

Height and weight were measured using a standard stadiometer and scale, with body mass index (kg/m^2^) was calculated from two averaged measurements. Waist and hip circumferences (to the nearest 0.5 cm) were measured using a plastic tape meter at the level of the umbilicus and of the greater trochanters, respectively, and waist-to-hip ratio was calculated. Blood pressure was measured with a standard mercury sphygmomanometer on the left arm after at least 10 min of rest. Mean values were determined from two independent measurements. Blood samples were collected after an overnight fast and analyzed for plasma glucose, insulin, serum total cholesterol, HDL-cholesterol, and triglycerides (LDL-cholesterol was estimated according to the Friedwald formula). Insulin resistance was estimated by the HOMA-IR index calculated from fasting glucose and insulin values (40). Subjects taking insulin were excluded from HOMA-IR analyses. Body composition of the subjects was estimated by dual-energy X-ray absorptiometry using a Hologic Horizon/A scanner (3-min whole-body scan, <0.1 G mGy) per manufacturer protocol. A single technologist analyzed all dual-energy X-ray absorptiometry measurements using Hologic Apex software (13.6.0.4:3) following the International Society for Clinical Densitometry guidelines. Visceral adipose tissue was measured in a 5-cm-wide region across the abdomen just above the iliac crest, coincident with the fourth lumbar vertebrae, to avoid interference from iliac crest bone pixels and matching the region commonly used to analyze visceral adipose tissue mass by CT scan (41).

### Human data statistical analysis

Normally distributed data were presented as mean ± standard deviation for continuous measures, and categorical data were expressed percentages. Unpaired independent Student’s *t* test was used to compare the differences between the two groups. One way analysis of variance was used to compare differences between groups. Categorical variables were compared using the Chi-squared test. Spearman rank correlation analysis was used to determine relationships among the independent variables and HOMA-IR. Multivariate linear regression models were fit for HOMA-IR as the dependent variable, and only variables significantly related (*p* < 0.05) to HOMA-IR by Spearman correlation were entered in the linear regression analysis. A two-sided value of *p* < 0.05 was considered statistically significant. All statistical analysis of the human data was performed using STATA version 15.1 (STATCorp LLC).

### Statistical analysis

One-way ANOVA was used to compare data between multiple groups followed by Bonferroni’s posttest. For analysis of blood glucose levels over time during glucose tolerance tests, a two-way ANOVA for repeated measures followed by Bonferroni’s posttest were used. All statistical analysis was performed using GraphPad Prism 9.0. Data are represented as mean ± SEM.

### Study approval

All experiments were approved by the Institutional Animal Care and Use Committee of UCSF and the UCSF Institutional Review Board.

## Data availability

All data are contained within the manuscript. Any additional information required to reanalyze the data reported in this paper is available from the lead contact Dr Kamran Atabai (Kamran.Atabai@ucsf.edu) upon request. This manuscript does not report any original code. This study did not generate any mass spectrometry data and/or PDB file.

## Supporting information

This article contains supporting information.

## Conflict of interest

The authors declare no conflict of interest with the contents of this article.
