# Supplementary material for: PTP1B mediates the inhibitory effect of MFGE8 on insulin signaling through the β5 integrin
Source: J Biol Chem. 2024 Jan 8;300(2):105631. doi: 10.1016/j.jbc.2024.105631 (PMC10850974; doi:10.1016/j.jbc.2024.105631)
Supplement: Supplementary Table 2 [file mmc1.docx]

| **Parameter** | **Spearman’s rho** | **P-value** |
| --- | --- | --- |
| Sex | 0.146 | 0.177 |
| Age (years) | 0.094 | 0.379 |
| Ethnicity | 0.271 | **0.010** |
| Weight, kg | 0.597 | **<0.001** |
| BMI, kg/m^2^ | 0.645 | **<0.001** |
| %Body Fat | 0.356 | **<0.001** |
| Systolic BP, mmHg | 0.306 | **0.004** |
| Diastolic BP, mmHg | 0.230 | **0.033** |
| Total Cholesterol, mg/dL | -0.069 | 0.522 |
| Triglyceride, mg/dL | 0.525 | **<0.001** |
| LDL, mg/dL | -0.122 | 0.261 |
| HDL, mg/dL | -0.386 | **<0.001** |
| HbA1c % | -0.503 | **<0.001** |
| Fasting glucose, mg/dL^a^ | 0.564 | **<0.001** |
| Insulin, mU/L^a^ | 0.931 | **<0.001** |
| Serum MFGE8, pg/mL | 0.221 | **0.038** |

**Supplemental Table 2: Correlation between HOMA-IR levels with demographic, body composition and biochemical characteristics.**

The relationships between HOMA-IR and various parameters were investigated using Spearman rank correlation. BMI, body mass index; BP, blood pressure; LDL, low-density lipoprotein cholesterol; HDL, high-density lipoprotein cholesterol; HbA1c, glycosylated hemoglobin; HOMA-IR (homeostasis model assessment of insulin resistance) = fasting insulin (mIU/L) × [fasting glucose (mg/dL)/405]; MFGE8, milk fat globule-epidermal growth factor. ^a^ Subjects on insulin were excluded from the analysis. Boldface *P* values are statistically significant (*P* < 0.05)
