# Supplementary material for: PTP1B mediates the inhibitory effect of MFGE8 on insulin signaling through the β5 integrin
Source: J Biol Chem. 2024 Jan 8;300(2):105631. doi: 10.1016/j.jbc.2024.105631 (PMC10850974; doi:10.1016/j.jbc.2024.105631)
Supplement: Supplementary Table 3 [file mmc2.docx]

| **Variable** | **Β coefficient** | **95% CI** | **p- value** |
| --- | --- | --- | --- |
| Ethnicity  Chinese  Hispanic | 0.9953148  1.008848 | .6899138,1.435906  .6662883,1.527528 | 0.980  0.966 |
| Weight, kg | 1.00279 | .9871913,1.018635 | 0.723 |
| BMI, kg/m^2^ | 1.006808 | .9598076,1.05611 | 0.777 |
| %Body Fat | 1.00497 | .9773017,1.033421 | 0.723 |
| Systolic BP, mmHg | 0.9943581 | .9822181,1.006648 | 0.359 |
| Diastolic BP, mmHg | 1.012484 | .9979501,1.02723 | 0.091 |
| HbA1c % | 1.004232 | .8610519,1.171221 | 0.956 |
| Triglyceride, mg/dL | 1.000612 | .9996838,1.00154 | 0.192 |
| HDL, mg/dL | 1.00158 | .9901064,1.013187 | 0.784 |
| Fasting blood glucose, mg/dL | 1.009417 | 1.003248,1.015625 | **0.003** |
| Fasting Insulin, mU/L | 1.024313 | 1.015021,1.03369 | **<0.0001** |
| Serum MFGE8, pg/mL | 1.000079 | 1.000004,1.000153 | **0.040** |

**Supplementary Table 3: Determinants of HOMA-IR in multivariate regression analysis**

Multivariate Linear regression analysis for the association of HOMA-IR as a dependent variable. BMI, body mass index; BP, blood pressure; HDL, high-density lipoprotein cholesterol; HbA1c, Hemoglobin A1c; HOMA-IR, homeostasis model assessment of insulin resistance; MFGE8, milk fat globule-epidermal growth factor. Boldface *P* values are statistically significant (*P* < 0.05)
