# Supplementary material for: PTP1B mediates the inhibitory effect of MFGE8 on insulin signaling through the β5 integrin
Source: J Biol Chem. 2024 Jan 8;300(2):105631. doi: 10.1016/j.jbc.2024.105631 (PMC10850974; doi:10.1016/j.jbc.2024.105631)
Supplement: Supplementary table 1 [file mmc3.docx]

|  | Lean (BMI≤25) (N=33) | Overweight/Obese (BMI>25) (N=56) | P-value |
| --- | --- | --- | --- |
| Male % (N) | 30.3 (10) | 48.2 (27) | 0.098 |
| Age, years | 41.67 (12.81) | 47.95 (10.99) | 0.016 |
| Ethnicity  White  Chinese  Hispanic | 12 (36.36%)  15 (45.45%)  6 (18.18%) | 19 (33.93%)  18 (32.14%)  19 (33.93%) | 0.24 |
| Weight, Kg | 61.07 (10.47) | 96.56 (24.32) | <0.001 |
| BMI | 21.97 (2.22) | 36.22 (8.18) | <0.001 |
| %Body Fat | 30.87 (6.06) | 39.37 (7.49) | <0.001 |
| Systolic BP | 123 (15.41) | 133.39 (13.11) | 0.001 |
| Diastolic BP | 71.91 (15.79) | 80.03 (10.81) | 0.006 |
| Hemoglobin A1c | 5.52 (0.66) | 6.53 (1.58) | 0.005 |
| Total Cholesterol, mg/dL | 189.55 (28.94) | 191.43 (49.31) | 0.84 |
| Triglyceride, mg/dL | 90.73 (50.37) | 148.91 (169.49) | 0.058 |
| LDL, mg/dL | 112.88 (28.08) | 115.31 (41.60) | 0.77 |
| HDL, mg/dL | 58.45 (13.28) | 49.00 (14.15) | 0.002 |
| HOMA-IR | 2.10 (1.79) | 9.27 (12.25) | 0.001 |
| Insulin, mU/L | 7.93 (3.80) | 28.47 (27.60) | <0.001 |
| Fasting glucose, mg/dL | 95.24 (16.67) | 117.41 (45.34) | 0.008 |
| Serum Mfge8, pg/mL | 2480.45 (2163.26) | 2463.58 (1809.93) | 0.97 |
| Insulin use (%) | 0 | 10 | 0.010 |
| Presence of T2D (%) | 1 (3.03) | 23 (41.07) | <0.001 |

**Supplementary table 1: Baseline Characteristics by BMI class.**

Data are presented as mean (SD) for continuous measures, and n (%) for categorical measures.
